# Suicide attempt risks among hotline callers with and without the coronavirus disease 2019 related psychological distress: a case-control study

**DOI:** 10.1186/s12888-021-03371-3

**Published:** 2021-07-20

**Authors:** Yongsheng Tong, Kenneth R. Conner, Yi Yin, Liting Zhao, Yuehua Wang, Mengjie Wu, Cuiling Wang

**Affiliations:** 1 Beijing Suicide Research and Prevention Center, Beijing Hui Long Guan Hospital, 7 Nan Dian Road, Changping, Beijing, 100096 China; 2 WHO Collaborating Center for Research and Training in Suicide Prevention, Beijing, China; 3 Peking University Huilongguan Clinical Medical School, Beijing, China; 4 Departments of Emergency Medicine and Psychiatry, University of Rochester Medical Center, Rochester, USA

**Keywords:** Suicide attempt, Coronavirus disease 2019, Hotline, Life events, Prior suicide attempt history, Moderator

## Abstract

**Background:**

The coronavirus disease 2019 (COVID-19) pandemic profoundly impacts on mental health, yet it is still unclear whether COVID-19 distress makes people more vulnerable to suicidal behavior. The present study aims to examine the association between COVID-19 related psychological distress and risk for suicide attempt, and moderators of this association, among hotline callers.

**Methods:**

This case-control study was conducted at the largest psychological support hotline in China. Hotline callers who sought help for psychological distress and reported whether or not they attempted suicide in the last 2 weeks (recent suicide attempt) were analyzed. The primary predictor of recent suicide attempt was the presence or absence of COVID-19 related psychological distress. Demographic variables and common risk and protective factors for suicidal behavior were also studied. Callers with COVID-19 related distress (COVID-19 callers) and those without such distress (non-COVID-19 callers) were compared on these variables. Recent suicide attempt was regressed on COVID-19 related distress and the other variables, and significant interaction terms of aforementioned predictors by COVID-19 related distress, to identify variables that moderate the association of COVID-19 related distress and recent suicide attempt.

**Results:**

Among 7337 included callers, there were 1252 COVID-19 callers (17.1%) and 6085 non-COVID-19 callers (82.9%). The COVID-19 callers were less likely to report recent suicide attempt (*n* = 73, 5.8%) than the non-COVID-19 callers (*n* = 498, 8.2%, *P* = 0.005). The COVID-19 callers were also less likely to have high scores on depressive symptoms (22.6% vs 26.3%, *P* < 0.001) and psychological distress (19.5% vs 27.3%, *P* < 0.001), and were more likely to have high hopefulness scores (46.5% vs 38.0%, *P* < 0.001). Tests of moderating effects showed that acute life events were associated with one-half lower risk (*P* = 0.021), and a trend that suicide attempt history was associated with two-thirds greater risk (*P* = 0.063) for recent suicide attempt, among COVID-19 callers than non-COVID-19 callers.

**Conclusions:**

The COVID-19 calls are from individuals with lower suicide-related risk compared to more typical callers. Acute stressful life events provided a key context for suicide attempt in non-COVID-19 callers, i.e., more typical calls.

**Supplementary Information:**

The online version contains supplementary material available at 10.1186/s12888-021-03371-3.

## Background

The coronavirus disease 2019 (COVID-19) outbreak began in December 2019 and has struck worldwide. Numerous studies reported that mental health symptoms were commonly reported among people during the COVID-19 pandemic [1–6]. Moreover, during the COVID-19 pandemic, suicide risk has been theorized to increase in the context of increased social isolation, economic stress, and barriers to treatment of mental illness, among other factors [7, 8]. Although we await definitive evidence of increased suicide rates during the pandemic [7, 9, 10], available data suggest that more patients presented to hospitals due to self-harm after the COVID-19 outbreak [11]. Previous online surveys of community samples reported that, during the pandemic, 5% of respondents reported a recent episode of self-harm [12], 7.6% of respondents were classified as high suicidal risk [13], and 8.8 to 25% reported a suicidal thought in the last week [14, 15]. Although these surveys did not include control groups, the high prevalence rates suggest elevated suicidal thoughts and acts (including suicide attempt and suicide death) during the COVID-19 pandemic. Moreover, the risk of self-harm or suicidal ideation has been shown to be associated with concerns about the pandemic and associated psychological distress [16, 17]. Although psychological distress and other suicide related risk factors appear to be increased during the COVID-19 pandemic, it remains unclear whether COVID-19 related distress is associated with increased risk for suicidal acts per se.

Due to the variety of measures employed to combat the COVID-19 pandemic such as lock-down, containment, community and school closure, internet and hotline resources have been widely used to collect data for mental health surveys and to deliver psychological interventions [18–20]. Google searches for distress helpline were remarkably elevated [21]. In China, the Guideline for Psychological Support Hotline was issued by the National Health Commission to standardize procedures and improve service quality of the hotline-based psychological intervention during the COVID-19 pandemic [22].

The Beijing Psychological Support Hotline provided psychological services to more than 169,000 callers in the first half-year of 2020. For each call made during the COVID-19 outbreak in China, the hotline operators were trained to classify it as a COVID-19 related call or non-COVID-19 related call (the criteria are described in detail in the Methods section). As a regular workflow, detailed information on suicide attempts and several common suicide risk factors were also assessed by the operator. In the present study, we compared the two groups of callers (COVID-19 and non-COVID-19) on demographic variables and common risk factors. We also examined risk for a recent suicide attempt (within 2 weeks of the index call) associated with COVID-19 related distress and common risk factors. Finally, we examined the potential moderating influence of COVID-19 related distress on the strength of association of these variables with risk for a recent suicide attempt. The results can inform the broader understanding of the role of COVID-19 related distress in suicide attempt as guide more specific telephone-delivered psychological intervention techniques and training.

## Methods

### Design, setting, and participants

The Beijing Psychological Support Hotline delivers psychological intervention to Mandarin speaking callers in China and overseas. In January 2020, at the beginning of the COVID-19 outbreak in China, all hotline operators received training for delivering hotline services during the outbreak. The training covered basic knowledge, information, policy, and facts about COVID-19, response to caller’s concerns and development of rapport with callers, providing emotional relief, problem identification, collaborative discussion, problem-solving skills, and crisis intervention for high suicidal risk callers. The intervention and training for callers was described in a recent study [23].

At the end of each call, the operator would classify whether the call was a COVID-19 related call based on the caller’s narration and the caller’s self-reported main problem. The criteria of “COVID-19 call” were: 1) the caller complains that he/she has been psychologically impacted by the COVID-19, or 2) the caller has encountered a problem due to the COVID-19, including oneself or family members infected by COVID-19, quarantine, lock-down, or unemployed due to unwanted closure. If a call met either criteria, the call would be classified as a COVID-19 call; else it would be classified as a non-COVID-19 call.

All calls from January 25th to June 15th 2020 were eligible. The exclusion criteria were as follows: 1) “invalid” calls, including silent or harassment calls, 2) calls lasting less than 10 min, 3) the caller’s main purpose was not seeking help for his/her psychological distress, 4) missing data on whether the callers had a “recent suicide attempt”, 5) repeated calls from the same caller. If more than one call of the same caller was eligible and enrolled, only the first call in the study period was included in the data analysis.

### Measures

Hotline operators conducted assessments at the beginning of a call. The operator asked the caller question(s) as follows, “Have you ever attempted suicide?”, if the caller responded “yes”, then “How many suicide attempts have you had in your life?” and “When did the latest suicide attempt occur?” A recent suicide attempt (main outcome in present study) was defined as suicide attempt occurred within 2 weeks before the call [24]. History of suicide attempts was defined as suicide attempts that occurred prior to 2 weeks before the incoming call.

While responding to a call, the hotline operators was required to rate and record the severity and the number of days of nine depressive symptoms of the caller presenting in the last 14 days before the incoming call., using the structured psychiatric examination [25]. This validated procedure yields a total score of depressive symptoms that is the sum of the product of severity and days for each depressive symptom [25]. The total score ranged from 0 to 100, with a higher score indicating more persistent and severe depressive symptoms. In the present study, the continuous depressive symptom score was converted into tertiles, i.e., mild (0–58), moderate (59–75), and severe (76–100).

Hopefulness and psychological distress were assessed by asking callers questions of “To what extent do you feel hopeful” and “To what extent do you feel psychological distress?” on scales from 0 to 100 (with higher scores indicating more hopeful and more distressed, respectively) using a validated procedure [23]. In the present study, the continuous scores of hopefulness and psychological distress were also converted into tertiles, i.e., hopeless (0), moderate hopeful (1–44) and high hopeful (45–100), and low stress (0–73), moderate stress (74–90) and severe stress (91–100).

The Beijing Psychological Support Hotline has established standardized hotline-based measurements for common suicide risk factors in Mandarin [24], with approximate English translations as follows. The presence of chronic life events was assessed by asking callers “In the last month, were you moderately or severely impacted by any long-term life events, including conflicts with family member(s), work disturbance, etc.?” If the caller responded “yes”, then it would be coded as “had chronic life events”. Similarly, the presence of acute life events was rated by asking the caller “In the last week, did there any negative life events occur and psychologically impact you?” If the caller responded “yes”, then it would be coded as “had acute life events”.

Substance misuse was assessed by asking callers “Have you excessively drank or been intoxicated at least four times in the last year and did it moderately or severely impact your mental health or disturbed you in the last month?” and “Have you excessively used addictive drugs for at least three consecutive months in the last year and did it moderately or severely impact your mental health or disturbed you in the last month?”. If the caller responded “yes” to any of the two questions, it would be coded as “had substance misuse”.

Fear of being attacked was assessed by asking callers “Did you moderately or severely fear being attacked in the last month?”. History of being maltreated was assessed by asking callers “Have you experienced physical or sexual abuse and were distressed by it or impacted moderately or severely in the last month?”. If the caller responded “yes” to any of the questions, it would be coded as “yes”, respectively.

Data were also collected on callers’ demographic variables, severe physical illness, and whether or not the blood-relatives, non-blood family members, or other acquaintances of the caller had a history of suicidal acts (suicide death or suicide attempts).

### Statistical analysis

Characteristics of the COVID-19 group and the non-COVID-19 group were compared using Chi-square test. A series of unadjusted logistic regression models were used to examine associations of demographic variables, common risk factors, and hopefulness (a protective factor) with a recent suicide attempt. Next, all demographic variables and risk and protective variables were entered into a multivariate logistic model. Finally, interaction terms between COVID-19 related distress and the other predictors were added to the logistic regression model and the backward wald method was used to select independent variables after demographic variables were adjusted [26]. We tested each interaction term one by one, and only the interaction terms with *P*-value less than 0.10 were considered in the final multivariate logistic regression model.

### Ethical approval and consent to participate

The study was approved by the institutional review board of the Beijing Huilongguan Hospital (2020–19-Science). All methods were performed in accordance with the Declaration of Helsinki and it’s later amendments. All participants in the present study, i.e., hotline callers, were fully informed by a voice message that all calls would be tape-recorded and data would be collected and analyzed anonymously, and informed consent were obtained from all paricipants. Before analysis, information about the callers was de-identified.

## Results

**Fig. 1:**
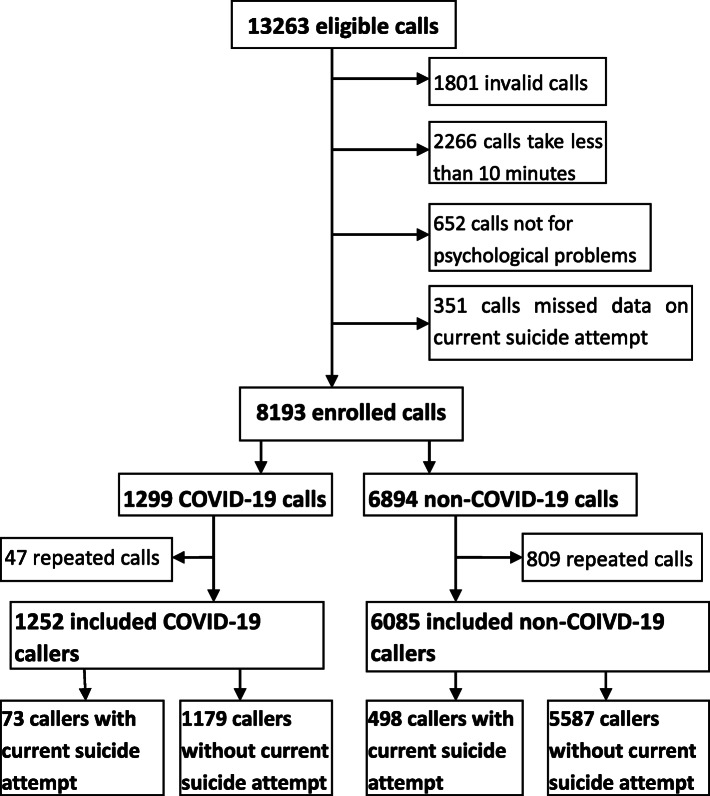
Flowchart of enrolling and screening callers

The detailed process of enrolling and screening hotline callers is shown in Fig. 1. Among 13,263 calls from January 25th to June 15th, 2020, 5070 calls were excluded. Among remained 8193 enrolled calls, 1299 were classified as COVID-19 calls and the other 6894 of them were non-COVID-19 calls. After 47 and 809 repeated calls of the two groups were excluded respectively, 7337 callers were included in the final data analysis. Seventy-three (5.8%) of the 1252 COVID-19 callers and 498 (8.2%) of the 6085 non-COVID-19 callers reported a suicide attempt within 2 weeks of the call (i.e., recent attempt).

The non-COVID-19 callers were statistically significantly younger than the COVID-19 callers ([22.1 ± 8.1] years vs [26.8 ± 11.4] years, t = 13.59, *P* < 0.001). As shown in the Table 1, the non-COVID-19 callers were more likely to attempt suicide in the last 2 weeks (8.2% vs 5.8%, *P* = 0.005) and to have a prior history of suicide attempt(s) (25.5% vs 21.0%, *P* < 0.001) than the COVID-19 callers. Furthermore, the non-COVID-19 callers were more likely to have severe depressive symptoms and psychological distress than the COVID-19 callers. Compared with non-COVID-19 callers, COVID-19 callers were more likely to be married, employed, and had higher education levels, were less likely to fear being attacked or having a history of being abused, and were more likely to score high on hopefulness.

**Table 1:** Characteristics of callers in the COVID-19 and non-COVID-19 groups

Variables	COVID-19 Group (*N* = 1252)		Non-COVID-19 Group (*N* = 6085)		χ^2^	*P* value
	n	%	n	%		
**Recent suicide attempt**	73	5.8%	498	8.2%	8.01	0.005
**Female**	822	65.7%	4101	67.4%	1.46	0.227
**Marital Status**					105.66	< 0.001
Unmarried	915	73.1%	5148	84.6%		
Married	260	20.8%	670	11.0%		
Cohabit	76	6.1%	238	3.9%		
**Work status**					62.37	< 0.001
Employed	507	40.5%	1979	32.5%		
Student	472	37.7%	3013	49.5%		
Unemployed	269	21.5%	1024	16.8%		
**Education level**					108.25	< 0.001
Primary school	87	6.9%	623	10.2%		
Middle school	443	35.4%	2901	47.7%		
College or university	702	56.1%	2435	40.0%		
**Depressive symptom score**					19.80	< 0.001
Mild	382	30.5%	1508	24.8%		
Moderate	299	23.9%	1484	24.4%		
Severe	283	22.6%	1598	26.3%		
**Psychological distress score**					34.57	< 0.001
Mild	411	36.7%	1653	30.2%		
Moderate	490	43.8%	2327	42.5%		
Severe	218	19.5%	1491	27.3%		
**Hopefulness score**					34.81	< 0.001
Hopeless	164	15.5%	1161	22.1%		
Moderate	400	37.9%	2099	39.9%		
High	491	46.5%	2001	38.0%		
**Substance misuse**	81	6.5%	399	6.6%	1.33	0.513
**Chronic life events**	632	50.5%	3148	51.7%	5.52	0.063
**Acute life events**	495	39.5%	2404	39.5%	2.38	0.304
**Being abused**	134	10.7%	833	13.7%	12.60	0.002
**Fear of being attacked**	144	11.5%	928	15.3%	17.84	< 0.001
**History of suicide attempts**	263	21.0%	1550	25.5%	16.13	< 0.001
**Physical illness**	94	7.5%	426	7.0%	1.62	0.444
**Relatives’ suicidal acts history**	406	32.4%	2051	33.7%	4.95	0.084

Because of data missing, in most variables, percentages don’t total 100%

**Fig. 2:**
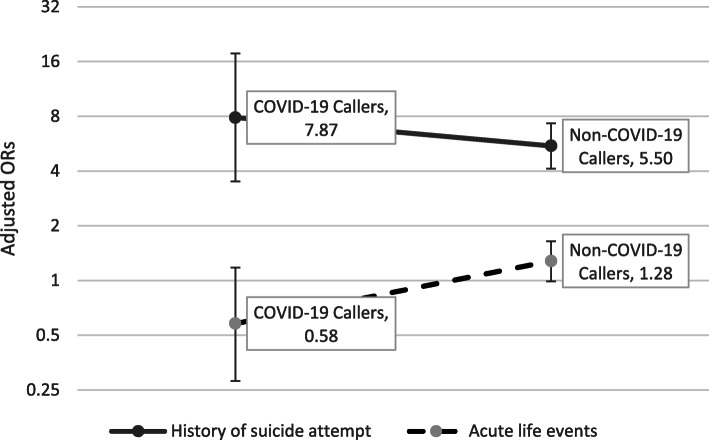
Comparisons of adjusted ORs of history of suicide attempt and acute life events for recent suicide attempt among hotline callers with and without COVID-19 related psychological disturbance. Age, gender, education level, marital and work status were adjusted for estimating the ORs

The Table 2 listed the risk of recent suicide attempt conveyed by different psychological and social factors among Chinese hotline callers with and without COVID-19 related distress. After adjusted for demographic variables, moderate or severe depressive symptoms, severe psychological distress, acute life events, substance misuse, and the caller’s suicide-attempt history increased the risk of recent suicide attempt, and high hopefulness reduced the risk of a recent suicide attempt to both groups of hotline callers. When interaction terms were added into the logistic regression model, the interaction of acute life events by COVID-19 reached statistical significance (*P* = 0.021), and the interaction of history of suicide attempt by COVID-19 was only a trend of statistical significance (*P* = 0.063). It implied that acute life events conferred a lower risk of recent suicide attempt to the COVID-19 callers than that to the non-COVID-19 callers, and the history of suicide attempts tended to confer a higher risk of a recent suicide attempt to the COVID-19 callers than that to the non-COVID-19 callers (see Fig. 2).

**Table 2:** Crude Odds Ratios (ORs) and adjusted ORs of common risk factors of suicide attempt among Psychological Support Hotline callers in China during the COVID-19 pandemic

Variables	Crude OR	95%CI	Adjusted OR^a^	95% CI	*P* value
**Age**	0.93	0.91–0.94	0.95	0.92–0.98	0.001
**Female**	1.69	1.38–2.07	1.37	1.03–1.82	0.031
**Marital status**					
Unmarried	1.00		1.00		
Married	0.44	0.31–0.62	1.59	0.95–2.66	0.077
Cohabit	0.82	0.53–1.28	2.14	1.17–3.92	0.014
**Work status**					
Employed	1.00		1.00		
Student	1.83	1.49–2.26	0.96	0.66–1.39	0.830
Unemployed	1.46	1.12–1.91	0.91	0.63–1.32	0.630
**Education level**					
Primary school	1.00		1.00		
Middle school	0.78	0.61–1.01	0.87	0.62–1.23	0.436
College or university	0.29	0.22–0.38	0.65	0.41–1.03	0.067
**Depressive symptom**					
Mild	1.00		1.00		
moderate	2.51	1.85–3.41	1.50	1.05–2.13	0.025
Severe	4.21	3.16–5.61	1.77	1.25–2.51	0.001
**Psychological distress**					
Mild	1.00		1.00		
Moderate	1.45	1.13–1.87	1.05	0.77–1.44	0.757
Severe	2.98	2.32–3.83	1.58	1.14–2.18	0.006
**Hopefulness**					
Hopeless	1.00		1.00		
Moderate	0.51	0.42–0.63	0.57	0.44–0.73	< 0.001
high	0.17	0.13–0.23	0.37	0.26–0.52	< 0.001
**Acute life events**	1.40	1.15–1.72	1.28	1.001–1.63	0.049
**Substance misuse**	2.60	2.00–3.38	1.46	1.07–2.00	0.017
**History of suicide attempts**	9.17	7.29–11.53	5.19	3.96–6.80	< 0.001
**Acute life events ×COVID-19 group**	/	/	0.47	0.24–0.89	0.021
**History of suicide attempt ×COVID-19 Group**	/	/	1.67	0.97–2.86	0.063
COVID-19 group	0.69	0.54–0.90	NS^b^		
Chronic life events	1.68	1.33–2.13	NS^b^		
Physical illness	1.07	0.77–1.49	NS^b^		
Being abused	1.56	1.24–1.97	NS^b^		
Fear of being attacked	1.84	1.47–2.29	NS^b^		
Relatives suicidal acts history	1.51	1.23–1.84	NS^b^		

The variables with *p* values greater than 0.10 in the multivariate logistic analysis were excluded from the final model

^a^Adjusted for age, gender, education level, marital and work status

^b^*NS* non-statistical significance

## Discussion

High rates of psychological distress during the COVID-19 pandemic is well-documented [1–6]. Adding to this corpus, the present study provides novel data on whether or not COVID-19 related distress is associated with risk for a recent suicide attempt in Chinese hotline callers, as well as how such distress may moderate (i.e., increase or decrease) suicide attempt risk associated with other with other predictors. Our results show that 17% (1252/7337) of non-repeat calls for psychological distress to the largest crisis hotline in China concerned COVID-19 related distress (COVID-19 callers), further evidence that online or hotline psychological services provide a critical outlet for individuals affected by the pandemic to receive support and intervention [18–21]. Indeed, more than one in 20 callers (5.8%) with COVID-19 related distress had attempted suicide within the past 2 weeks, underscoring that callers to the crisis line with COVID-19 related distress are a high-risk population. However, contrary to our hypothesis, these callers showed somewhat lower likelihood of suicide attempt within the past 2 weeks than non-COVID-19 callers (8.2%). Moreover, COVID-19 callers showed fewer common risk factors for suicidal acts including lower levels (or lower likelihood) of lifetime suicide attempt(s), depressive symptoms, psychological distress, and abuse history. COVID-19 callers were also more likely to score high on hopefulness and to a have range of demographic characteristics that are protective from attempted suicide including being married, employed, and higher education. The findings indicated that calls prompted by COVID-19 related distress were made by callers at lower risk for suicide attempt than calls that were not prompted by such distress.

Our results showed that individuals making calls to crisis line with COVID-19 related distress had somewhat more protective factors from suicidal acts, lower risk factors for suicidal acts, and were at lower risk for a recent suicide attempt than other callers. A potential explanation is that COVID-19 related distress is protective from suicide attempt, with potential mechanisms including that it may promote a sense of connectedness to others under a common threat including harmony within families [7, 27]. However, on balance this idea seems implausible in light of theorized risks with the COVID-19 pandemic related to social isolation, economic hardship, and other factors [8], along with data in general samples showing elevated rates of psychological distress and suicidal thoughts or behavior during the pandemic [1–6, 11–15, 28]. In our view, a more satisfactory explanation is that COVID-19 related distress prompted calls to the crisis line by individuals who are at generally lower risk for suicide attempt than typical hotline callers, a high-risk population who are likely to contact the crisis line in the context of other acute stressors (e.g., interpersonal distress) [29, 30]. In support of this idea, a test of moderation (i.e., statistical two-way interaction) showed that, after demographic variables were adjusted, the strength of association between a recent suicide attempt and experiencing acute life event(s) was weaker in COVID-19 callers than that in non-COVID-19 callers (*P* = 0.021). The decrease of the association strength might be attributed to the features of acute life events the COVID-19 callers encountered, e.g., transient work or financial problems due to quarantine or lock down which was removed soon.

One other test of moderation also bears discussion, with callers with a history of suicide attempt showing a trend to be more likely to make a recent suicide attempt in the context of COVID-19 related distress (*P* = 0.063). The current investigation did not attempt to parse out types of COVID-19 related distress, but prior studies suggest that social isolation and limited resources associated with containment efforts may be particularly stress inducing [31, 32], whereas physical distancing in and of itself may not be suicidogenic [16]. Demographic variables, socioeconomic status, and social resources could also moderate the risk for suicidal behavior during the COVID-19 pandemic [12, 14, 17]. Altogether, the results underscore the complex nature of the relationship between COVID-19 related distress and suicidal acts, including the critical importance of considering moderating effects and the population under study. Along these lines, our result of lower risk for suicide attempt among individuals with COVID-19 related distress was identified in a high-risk sample of callers to a suicide hotline and would not expect to be found in a general sample.

There are several limitations in the present study. First, all callers were exposed to the COVID-19 pandemic at some level, and our measure of COVID-19 related psychological distress does not imply the complete absence of exposure. Second, all information, including episodes of suicide attempt, was self-reported and collected via telephone. Third, the participants of our study were hotline callers who seeking help for psychological distress and, as we have discussed, due to the unique demographic characteristics of hotline callers in our study, generalization to other populations is unclear, particularly lower risk samples. Fourth, information on whether or not the callers were infected by COVID-19, were quarantined, or were in contact with confirmed cases were not collected. Fifth, we didn’t detect the changes of the associations at different stages of the pandemic. Sixth, the data missing in most variables might weaken validity of our findings. Finally, the majority of the participants of the present study were recruited in mainland China, where the COVID-19 epidemic was mitigated effectively after about 2 months (February and March), a much shorter period of outbreak than many countries.

## Conclusions

To our knowledge, this study of callers to the largest suicide hotline in China during the COVID-19 pandemic is the first study to examine risk for recent suicide attempt associated with COVID-19 related distress in a hotline sample, and the first to explore moderators of the association between COVID-19 related distress and risk for recent suicide attempt. Results suggest that COVID-19 related distress may prompt calls to the crisis line among individuals who are generally at lower risk for suicidal act than typical callers. However, the potential moderation of COVID-19 related distress on associations of suicide attempt and acute life events and history of suicide attempt underscore the importance of outreach and intervention with these vulnerable individuals during the pandemic.

## Supplementary Information

**Additional file 1.**

## Data Availability

The dataset used and analyzed during this study are available from the corresponding author on reasonable request.

## Abbreviation

COVID-19: Coronavirus disease 2019
