# Supplementary material for: Suicide attempt risks among hotline callers with and without the coronavirus disease 2019 related psychological distress: a case-control study
Source: BMC Psychiatry. 2021 Jul 20;21:363. doi: 10.1186/s12888-021-03371-3 (PMC8290867; doi:10.1186/s12888-021-03371-3)
Supplement: Supplementary file 1 — Additional file 1. [file 12888_2021_3371_MOESM1_ESM.doc]

**Suicide attempt risks among hotline callers with and without the coronavirus disease 2019 related psychological distress: a case-control study**

Yongsheng Tong^1,2,3,*^, Kenneth R. Conner ^4^, Yi Yin^1,2,3^, Liting Zhao^1,2^, Yuehua Wang^1,2^, Mengjie Wu^1,2,3^, Cuiling Wang^1,2^

^1^ Beijing Suicide Research and Prevention Center, Beijing Hui Long Guan Hospital

^2^ WHO Collaborating Center for Research and Training in Suicide Prevention

^3^ Peking University Huilongguan Clinical Medical School

^4^ Departments of Emergency Medicine and Psychiatry, University of Rochester Medical Center

***Corresponding author**: Yongsheng Tong, PhD, Beijing Suicide Research and Prevention Center, Beijing Huilongguan Hospital, 7 Nan Dian Road, Changping, Beijing, 100096, China (timystong@pku.org.cn)

**The questionnaire and interviews have been cited in the manuscript (references 23, 24, 25)**

**Depression**

| **1** | (a) Have you felt depressed in the past two weeks? | **Yes** | | | | | | **No [Skip to 2]** | | | |
| --- | --- | --- | --- | --- | --- | --- | --- | --- | --- | --- | --- |
|  | (b)How long have you been consistently in such feeling, most of the day, almost every day? | **days** | | | | | | | | | |
| **2** | **(a)**In the past two weeks, have you been uninterested in most things or unable to enjoy the things you used to enjoy in most time? | **Yes** | | | | | | **No**  **[Skip to 3]** | | | |
|  | **(b)**How long have you been consistently drown in such feeling almost every day? | **days** | | | | | | | | | |
| **3** | **(a)**In the past two weeks, have you repeatedly considered hurting yourself, feeling suicidal, or want to die? | **Yes**  **[Skip to (c1)]** | | | | | | **No** | | | |
|  | **(b)**In the past two weeks, have you repeatedly felt tired, hard, boring of living, even would rather to die? | **Yes** | | | | | | **No**  **[Skip to (d1)]** | | | |
|  | **(c1)**In the past two weeks, have you ever had a specific suicide plan?  [If Yes, ask subjects to describe the specific situation. ] | **Yes** | | | | | | **No** | | | |
|  | **(c2)**If "0" means not at all, and "100" means completely, to what extent did you want to die？ | **(0-100)** | | | | | | | | | |
|  | **(d1)**Have you ever attempted suicide or hurt yourself?  [If Yes, ask callers to describe the specific situation, feeling, and current attitude to the suicide attempt. ] | **Yes (Describe:**  **）** | | | | | | **No**  **[Skip to (e)]** | | | |
|  | **(d2)**How many suicide attempts have you had in your life? | **Times** | | | | | | | | | |
|  | **(d3)**When did the latest suicide attempt occur? | **/ /**  **day/month/year** | | | | | | | | | |
|  | **(d4)**Where did the latest suicide attempt occur?  1=Home 2=Workplace 3=Garden 8=Other |  | | | | | | | | | |
|  | **(d5)**What kind of method of suicide attempt did you take?  1=Self-poisoning 2=Take medication 3=Cutting  4=Hanging/Suffocation 5=Fall down 8=Other |  | | | | | | | | | |
|  | **(d6)**What was the main reason for your suicide?  01=Romance relationship problems 02=Family disputes  03=Other interpersonal conflicts 04=Work/study problems 05=Financial difficulties 06=Other financial problems  07=Physical illness 08=Depressed  09=Alcohol use problems 10=Other mental problems 11=Supernatural related problems 88=Other | **___** | | | | | | | | | |
|  | **(d7)**What was the main purpose for your suicide attempt?  1=Release the burden on others 2=Relieve psychological distress  3=Fight against encounters 4=Avoid responsibility 5=Revenge related persons 6=Threat to others 8=Other |  | | | | | | | | | |
|  | **(d8)**If "0" means not at all, and "100" means completely want to die, to what extent did you want to die？ | **(0-100)** | | | | | | | | | |
|  | **(d9)**Were you resuscitated by medical personnel after attempted suicide? | **Yes** | | | | | | **No** | | | |
|  | **(e)**[Investigator judgment] About subjects’ suicide plan recently：  1= No suicidal ideation  2= Have suicidal ideation, without suicide plan  3= Not implementing suicide plan recently (within 1 week)  4= Possibly implementing suicide plan recently (within 1 week)  5= Suicide plan will be carried out  6= Suicide attempt in last 2 weeks  7= Ongoing suicidal action |  | | | | | | | | | |
| **4** | **(a1)**In the past two weeks, have you gained or lose weight than normal? | | **Yes** | | | | | | **No**  **[Skip to (b1)]** | | |
|  | **(a2)**Did you put on or reduce weight deliberately？ | | **Yes [Skip to (b1)]** | | | | | | **No** | | |
|  | **(a3)**Did your weight decrease or increase? | | **Decrease** | | | | | | **Increase** | | |
|  | **(a4)**Could others recognize the change of your weight? | | **Yes** | | | | | | **No [Skip to (b1)]** | | |
|  | **(a5)**How long have you been consistently in this condition? | | **Day**  **[If ≥14days, skip to 5]** | | | | | | | | |
|  | **(b1)**In the past two weeks, did your appetite change than normal? | | **Yes** | | | | **No**  **[Skip to (c1)]** | | | | |
|  | **(b2)**Did you try to gain or lose weight deliberately？ | | **Yes [Skip to (c1)]** | | | | **No** | | | | |
|  | (**b3)**Was your appetite decreased or increased? | | **Decrease** | | | | **Increase** | | | | |
|  | **(b4)**How long have you been consistently in this condition nearly every day? | | **Day**  **[If ≥ 14days, skip to 5]** | | | | | | | | |
|  | **(c1)**In the past two weeks, have you forced yourself to eat? | | **Yes** | | | | **No**  **[Skip to 5]** | | | | |
|  | **(c2)**How long have you been consistently in this condition nearly every day? | | **Day** | | | | | | | | |
| **5** | **(a)**In the past two weeks, have you ever had trouble on sleeping nearly every night (difficulty falling asleep, waking up in the midnight, early wakening or sleeping excessively)? | | **Yes** | | | | **No**  **[Skip to 6]** | | | | |
|  | **(b)**Did your sleeping time decrease or increase? | | **Decrease** | | | | **Increase** | | | | |
|  | **(c)** How many hours does your sleeping time decrease or increase, compared with normal? | | **___Hour/ Minute** | | | | | | | | |
|  | **(d)**How long have you been consistently in this condition nearly every day? | | **days** | | | | | | | | |
| 6 | **(a1)**In the past two weeks, have you ever felt agitation, restless or difficult to sit around, which could be noticed by others? | | **Yes** | | | | **No [Skip to (b1)]** | | | | |
|  | **(a2)**How long have you been consistently in this condition nearly every day? | | **days**  **[If ≥ 14days, skip to 7]** | | | | | | | | |
|  | **(b1)**In the past two weeks, have you ever talked or moved more slowly than normal, which could be noticed by others? | | **Yes** | | | | **No**  **[Skip to 7]** | | | | |
|  | **(b2)**How long have you been consistently in this condition nearly every day? | | **days** | | | | | | | | |
| **7** | **(a)**In the past two weeks, have you ever felt tired or without energy almost every day? | | **Yes** | | | | **No**  **[Skip to 8]** | | | | |
|  | **(b)**How long have you been consistently in this condition nearly every day? | | **days** | | | | | | | | |
| **8** | **(a1)**In the past two weeks, have you felt worthless? | | **Yes** | | | | **No [Skip to (b1)]** | | | | |
|  | **(a2)**How long have you been consistently in such condition, nearly every day? | | **days**  **[If ≥ 14days, skip to 9]** | | | | | | | | |
|  | **(b1)**In the past two weeks, did you feel guilty about what you have done or even what you haven’t done? | | **Yes** | | | | **No**  **[Skip to 9]** | | | | |
|  | **(b2)**How long have you been consistently in such condition, nearly every day? | | **Day** | | | | | | | | |
| **9** | **(a)**In the past two weeks, have you had difficulty concentrating, thinking or making decisions about daily matters? | | **Yes** | | | **No**  **[Skip to 10]** | | | | | |
|  | **(b)**How long have you been consistently in this condition, nearly every day? | | **days** | | | | | | | | |
| **10** | **(a)**In the past two weeks, did you experience a loss of libido? | | **Yes** | | | **No**  **[Skip to 11]** | | | | | |
|  | **(b)**In the past two weeks, to what extent did these problems (Name the symptoms admitted above) affect you on the following aspects respectively ? | | **days** | | | | | | | | |
| **11** | In the past two weeks, to what extent did these problems (Name the symptoms admitted above) affect you on the following aspects respectively ? | | | | | | | | | | |
|  | ① Work/Study ability | | None | Mild | Moderate | | | | | Severe | Extremely Severe |
|  | ② Daily life | | None | Mild | Moderate | | | | | Severe | Extremely Severe |
|  | ③ Mental health | | None | Mild | Moderate | | | | | Severe | Extremely Severe |
|  | ④ Social interaction | | None | Mild | Moderate | | | | | Severe | Extremely Severe |
|  | ⑤ Self care | | None | Mild | Moderate | | | | | Severe | Extremely Severe |
| **12** | In the past two weeks, to what extent did these problems make you feel distress? None, mild, moderate, severe, or extremely severe? | | None | Mild | Moderate | | | | | Severe | Extremely Severe |

**Other interviews**

| **13** | **(a)**Have you had repeatedly drunk (at least 4 times), difficulties in work or study, conflicts with others, consistently agitated or physical illness due to excessive drinking in the last year？ | **Yes** | | | **No**  **[Skip to 14]** | | |
| --- | --- | --- | --- | --- | --- | --- | --- |
|  | **(b)**Did these problems still exist in the last month? | **Yes** | | | **No**  **[Skip to 14]** | | |
|  | **(c)**In the last month, to what extent did these problems make you feel distress and annoyed, or interfere with your normal routine, work or study, usual social activities and relationships? | None | Mild | Moderate | | Severe | Extremely Severe |
| **14** | **(a)**Have you excessively used addictive drugs (hypnotics, tranquilizers, narcotics, stimulants, etc. ) for at least three consecutive months in the last year or have you ever had street drugs before? | **Yes** | | | | **No**  **[Skip to 15]** | |
|  | **(b)**Did these problems still exist in the last month? | **Yes** | | | | **No**  **[Skip to 15]** | |
|  | **(c)**In the last month, to what extent did these problems make you feel distress and annoyed, or interfere with your normal routine, work or study, usual social activities and relationships? | None | Mild | Moderate | | Severe | Extremely Severe |

| **15** | Do you have any physical illness or disability that has a serious impact on your life? | **Yes** | | | **No** | | | |
| --- | --- | --- | --- | --- | --- | --- | --- | --- |
| **16** | **(a)**In the last month, were you impacted negatively by any chronic life events, including conflicts with family member(s), work disturbance, etc.?  [If Yes, ask subjects to describe the specific situation. ] | **Yes (Describe:**  **）** | | | **No**  **[Skip to 17]** | | | |
|  | **(b)**To what extent did this problem impact you in the last month? | Mild | Moderate | | Severe | | Extremely Severe | |
| **17** | **(a)**In the last week, did there any acute negative life events occur and psychologically impact you? | **Yes** | | | **No**  **[Skip to 18]** | | | |
|  | **(b)**To what extent did this issue affect you in the last month? | Mild | Moderate | | Severe | | Extremely Severe | |
|  | **(c)**How many days have this problem impacted on you this week? | **days** | | | | | | |
| **18** | **(a)**Did you experience physical or sexual abuse before? | **Yes** | | | **No**  **[Skip to 19]** | | | |
|  | **(b)**[Simply describe this issue ] At what age you firstly experienced this? | **Describe:** | | | **__ __Years** | | | |
|  | **(c)**When did it the last time occur? | **Month/**  **Year** | | | | | | |
|  | **(d)**To what extent did this issue affect you in the last month? None, mild, moderate, severe, or extremely severe? | None | Mild | Moderate | | Severe | | Extremely Severe |
| **19** | **(a)**Did you fear being attacked in the last month? | **Yes** | | **No**  **[Skip to 20]** | | | | |
|  | **(b)**[Simply describe this issue ] To what extent did you fear being attacked in the last month? |  | |  | | | | |
|  | **Describe：** | None | Mild | Moderate | | Severe | | Extremely Severe |
|  | **(c)**How long has this problem been going on? | **days** | | | | | | |
| **20** | **(a)**How many of your blood-relatives have ever attempted suicide or died by suicide? [If No, write down the number 00.] | **Persons** | | | | | | |
|  | **(b)**How many people who you were familiar with, or your non-blood-relatives, have ever attempted suicide or died by suicide?  [If No, write down the number 00.] | **Persons** | | | | | | |
| **21** | If "0" means not at all, and "100" means the most hopeful, to what extent do you feel **hopeful** for future life**？** | **(0-100)** | | | | | | |
| **22** | If "0" means not at all, and "100" means completely, to what extent do you feel psychological **distress**? | **(0-100)** | | | | | | |
